# Influence of ulnar bow sign on surgical treatment of missed Bado type I Monteggia fracture in children

**DOI:** 10.1038/s41598-022-14513-2

**Published:** 2022-06-17

**Authors:** Shijie Liao, Tiantian Wang, Qian Huang, Yun Liu, Rongbin Lu, Yaofeng Xu, Xiaofei Ding

**Affiliations:** 1 Department of Orthopedics, The First Affiliated Hospital of Guangxi Medical University, 6 Shuangyong Road, Nanning, Guangxi China; 2 Research Centre for Regenerative Medicine, Guangxi Key Laboratory of Regenerative Medicine, Guangxi Medical University, 22 Shuangyong Road, Nanning, Guangxi China; 3 Department of Orthopedics, The First People’s Hospital of Nanning, Nanning, 530000 Guangxi China

**Keywords:** Trauma, Musculoskeletal system

## Abstract

The present study aimed to explore the influence of ulnar bow on the surgical treatment of Bado type I missed Monteggia fracture in children. A retrospective review of 28 patients was conducted between November 2010 and June 2020. All patients were treated with open reduction of the radial head and ulnar opening wedge osteotomy without annular ligament reconstruction. Four months (range 1–12 months) was the mean interval between injury onset and surgery. The average age of patients at the time of surgery was 6.1 years old (range 2–10 years old). The maximum ulnar bow (MUB) and MUB position (P-MUB) via radiography were evaluated. The patients were divided into two groups according to P-MUB, as follows: middle group (A) included 17 cases, and the MUB was located at 40–60% of the distal ulna; and distal group (B) included 11 cases, and the MUB was located at 20–40% from the distal end of the ulna. The mean follow-up period was 33 months (range 6–102 months). At the last follow-up, all the children showed stable reduction of the radial head, and the flexion function of elbow joint improved after the operation (P < 0.05). Group A presented a larger ratio of maximum ulnar bow (R-MUB) and angle of ulnar osteotomy (OA) than group B (P < 0.05). The osteotomy angle was positively correlated with the R-MUB (R^2^ = 0.394, P = 0.038). The osteotomy angle was positively correlated with the P-MUB (R^2^ = 0.683, P = 0.000). The R-MUB was proportional to the P-MUB (R^2^ = 0.459, P < 0.0001). The regression equation of P-MUB and osteotomy angle was as follows: OA = 32.64* P-MUB + 7.206. If the ulnar bow was positioned at the middle ulna, then a stable reduction of radial head needed to be achieved through a large angle in the ulnar osteotomy. If the position of maximum ulnar bow (P-MUB) was closer to the middle of the ulna, or the ratio of maximum ulnar bow (R-MUB) was larger, then the osteotomy angle was larger.

## Introduction

Missed Bado type I Monteggia fracture is characterized by an arcuate curvature of the metacarpal side of the ulna and anterior dislocation of the radial head. Bado type I is the most commonly missed Monteggia fracture that easily causes elbow bulge or cubitus valgus deformity, limited elbow movement, joint instability, joint pain of different degrees, and secondary nerve paralysis^1–3^. Therefore, active surgical treatment is recommended. Humeroradial joint open reduction and ulnar opening wedge osteotomy with or without annular ligament reconstruction are currently the preferred clinical treatments for missed Monteggia fracture in children^4^.

However, radial head re-dislocation and forearm rotation limitation are still the most common complications in the surgical treatment of neglected Monteggia lesion^2^. Angulation in the opposite direction after ulnar osteotomy is an effective measure to prevent postoperative complications^5–9^.

In 1994, Lincoln^10^ proposed the concept of ulnar bow sign, which indicates serious injury to the forearm. For missed Monteggia fracture, the ulna has basically healed; the bow sign is an important factor that can be used to measure the injury condition at this point. Hoon Park et al.^11^ pointed out that the ulnar bow sign can be used as an indicator of surgical strategy formulation. Stable reduction could be achieved through simple incision of radiocapitellar joint when the maximum ulnar bow is less than 3 mm and located at 40% of the distal ulna.

A study^11^ reported the relationship of ulnar bow sign with the indication for ulnar osteotomy. However, few papers have explored the influence of the relationship between ulnar bow sign and ulnar osteotomy angle on the operation. The present study aimed to provide a predictive index for the preoperative planning, surgical efficacy, and complication prevention in missed Monteggia fracture.

## Methods

**Figure 1:**
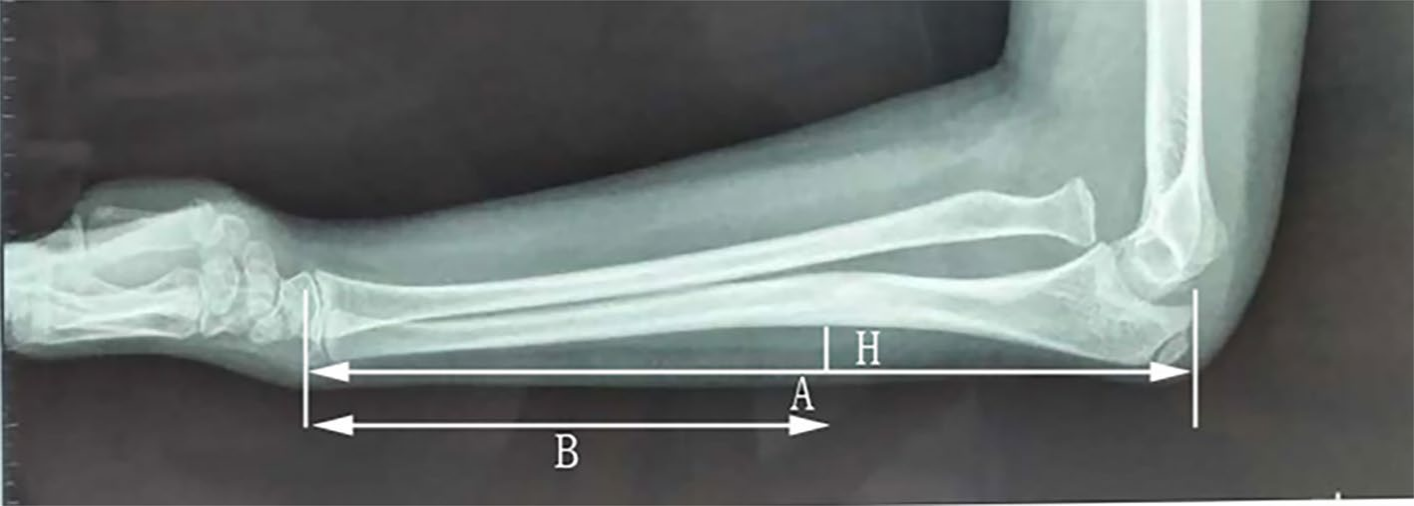
A line is drawn from the olecranon to the metaphysis of the distal end of the ulna. The maximum vertical distance from the straight line to the dorsal edge of the ulna is the maximum arcuate distance of the ulna and is recorded as MUB (H), A is the distance from the olecranon to the epiphysis of the distal ulna, and B is the distance from the position of the maximum arcuate sign to the epiphysis of the distal ulna. The maximum bow ratio is recorded as R-MUB (H/A), and the position of the maximum bow distance is recorded as P-MUB (B/A).

From November 2010 to June 2020, 28 children with Bado type I missed Monteggia fracture were treated by open radial head reduction and ulnar osteotomy without annular ligament reconstruction. Twenty males and 8 females, which included 11 left-sided and 17 right-sided fractures, were enrolled in the present study. The average time from injury to operation was 5 months (range 1–12 months). The average age of children who underwent the operation was 6.1 years old (range 2–10 years old), and the average follow-up time was 33 months (range 6–102 months). Most of the children included the study consulted with a doctor because of elbow flexion and extension dysfunction or the presence of a hard mass in front of the elbow. Among these patients, two cases had 30°–35° cubitus valgus deformity, and three cases featured a deep branch of radial nerve injury and limited wrist and thumb extension function without skin sensory disturbance. According to the full-length lateral radiograph of the forearm, the radial head was dislocated forward, and the ulna was curved to the palmar side, thereby indicating a ulnar bow sign. On the lateral radiograph of the forearm, a straight line was made between the olecranon and the distal ulnar metaphysis. The maximum vertical distance between the dorsal edge of the ulna and the straight line was defined as the maximum ulnar bow (MUB). To eliminate the influence of different X-ray proportion, the size of MUB by the ratio of the maximum bow distance was compared with the ulnar length (MUB ratio, R-MUB). Subsequently, the ratio of the distance from the largest arch to the distal ulna to the ulnar length (P-MUB) was used to express the position of MUB (Fig. 1).

The patients were divided into two groups according to the P-MUB, as follows: the middle group (A) comprised 17 cases (40–60% of P-MUB in distal ulna); and the distal group (B) comprised 11 cases (20–40% of P-MUB in distal ulna). We measured the MUB, ulnar angulation angle during operation, radial head stability after osteotomy, and elbow joint function (Table 1).

**Table 1:** Data of Group A and B.

	Group A	Group B	No. of patients	P value
**Sex**				
Male	12	8	20	–
Female	5	3	8	
**Side**				
Left	8	3	11	–
Right	9	8	17	
Age at Surgery (yr)	5.77 ± 2.61	6.73 ± 1.85	6.14 ± 2.35	0.299
Delay to surgery (mo)	5.12 ± 3.30	4.64 ± 2.84	4.93 ± 3.08	0.694
Follow-up time (mo)	35.29 ± 24.74	29.10 ± 17.13	32.86 ± 21.93	0.475
P-MUB	0.50 ± 0.07	0.30 ± 0.07	0.42 ± 0.12	–
R-MUB	0.041 ± 0.017	0.028 ± 0.011	0.036 ± 0.016	0.032

*P-MUB* the position of maximum ulnar bow, *MUB* the ratio of maximum ulnar bow.

### Surgical technique

All patients were treated by using combined anterior and posterior approach^12^, and the open reduction of radial head and fibrous scar resection were treated by using anterior Henry’s approach. The posterior approach was used for ulnar angulation and lengthening. For the anterior Henry’s approach, we performed a skin incision parallel to the forearm that extended to 4–6 cm along the elbow. We cut along the gap between biceps brachii tendon and brachioradialis brachii muscle, after which we then recognized and protected the radial nerve in the deep muscle gap. Three children were found to have radial nerve palsy by preoperative examination. It was found intraoperatively that the radial nerve became thinner and pale, and neurolysis was performed at the same time. After exposing the annular ligament, the radial head was dislocated and covered with fibrous scar tissue, which was subsequently removed. Proximal ulnar opening wedge osteotomy was performed by using the posterior approach. The proximal part of the ulna was exposed through a 6 cm- to 8 cm-long incision on the ulnar side, and the transverse osteotomy was performed 4–5 cm below the ulnar olecranon. Afterward, we pressed the radial head backward under the direct vision of the anterior approach to achieve a full reset. We rotated the forearm and repositioned the radial head to guide the final position of the ulna. The radial head was reduced, and stability was evaluated. The reduction of the radial head was dynamically observed through the anterior approach under different conditions of elbow flexion and extension and forearm rotation, especially the stability of the radial head under full extension of the elbow and full forearm rotation. K-wire was used temporarily for cases with unstable radial head reduction. Osteotomy was fixed with a pre-curved plate. C-arm fluoroscopy was used to confirm the position of radial head and the correct fixation of the plate and screw. In our preoperative evaluation, iliac bone transplantation was performed if the patient required a large Angle wedge osteotomy and lengthening of the ulna greater than 1 cm.

### Postoperative management

The elbow joint was immobilized in flexion position and forearm neutral position or supination position by long arm plaster cast. Approximately 3–6 weeks after the operation, the anteroposterior and lateral X-ray results of the elbow joint were reviewed, the K-wire and the plaster cast were removed. Periodic re-examination was conducted to monitor the possible occurrence of redislocation and functional recovery. Approximately 6–12 months after the operation, the steel plate was removed when the X-ray showed the bony healing of the osteotomy end.

### Statistical analysis

SPSS 19.0 was used for statistical analysis. We compared the flexion and extension of elbow joint and the rotation range of the forearm before and after operation via paired *t* test. Independent sample *t* test and Chi-square test were used to compare the two groups. Parameters of the two groups include Sex, Side, Age at Surgery, Delay to surgery, Follow-up time. Linear regression was used to analyze the factors affecting the osteotomy angle. P ≤ 0.05 was considered statistically significant.

### Ethics approval and consent to participate

We confirmed that all methods were carried out in accordance with relevant guidelines and regulations. We confirm that all experimental protocols were approved by Ethics Committee of the First Affiliated Hospital of Guangxi Medical University. The informed consent was obtained from parents of all participants, and publication of identifying images in an online open-access publication.

## Results

**Figure 2:**
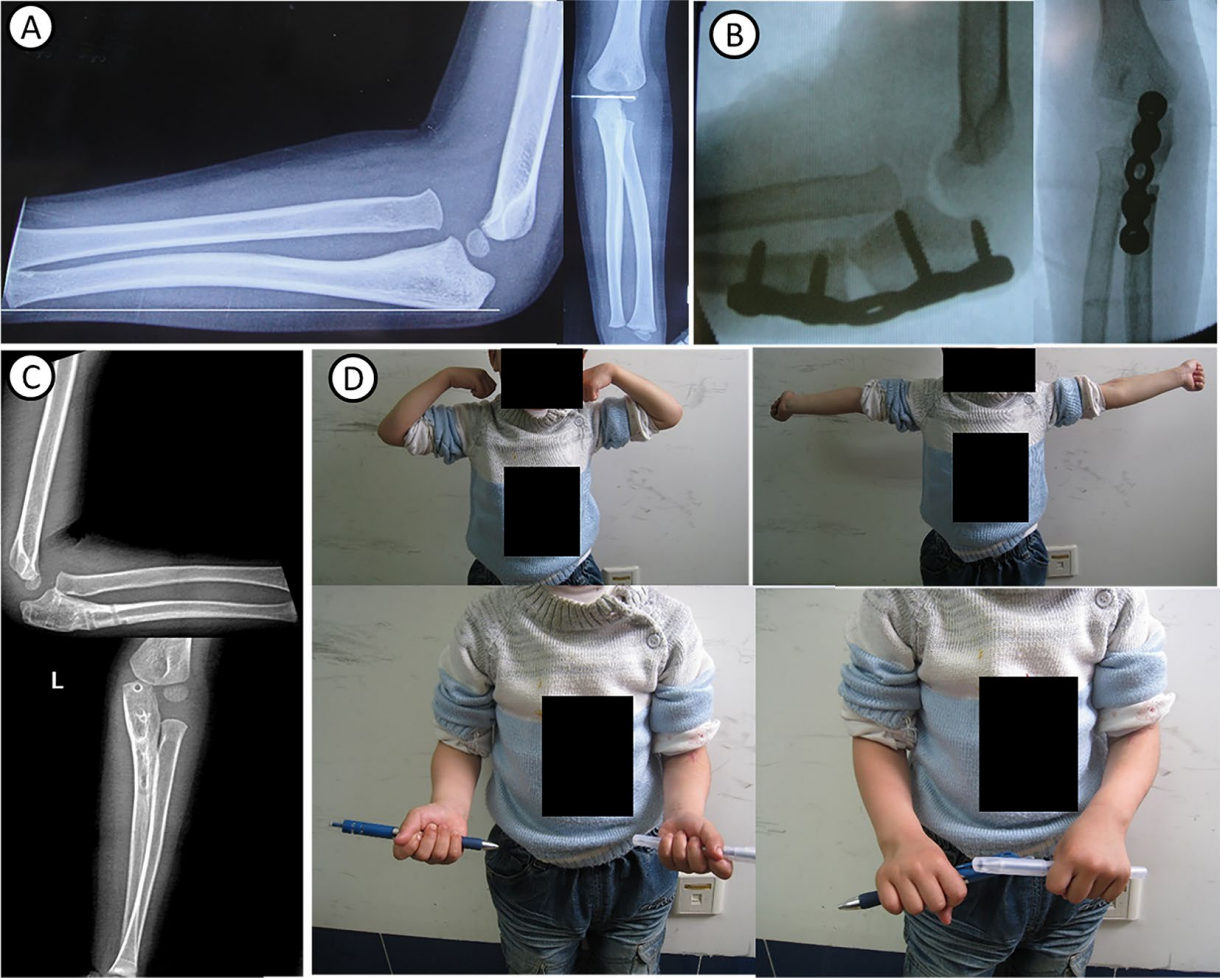
Typical case 1: A 3.5-year-old boy with left missed Monteggia fracture. (**A**) Bado I type, ulna bow sign is located in the middle of ulna (group A). (**B**) In operation, reverse angulation is 24°, and radial head reduction is observed. (**C**) No dislocation of the radial head is found after the osteotomy is healed and the internal fixation is removed. (**D**) The elbow joint function is good after operation, no loss of rotation function.

**Figure 3:**
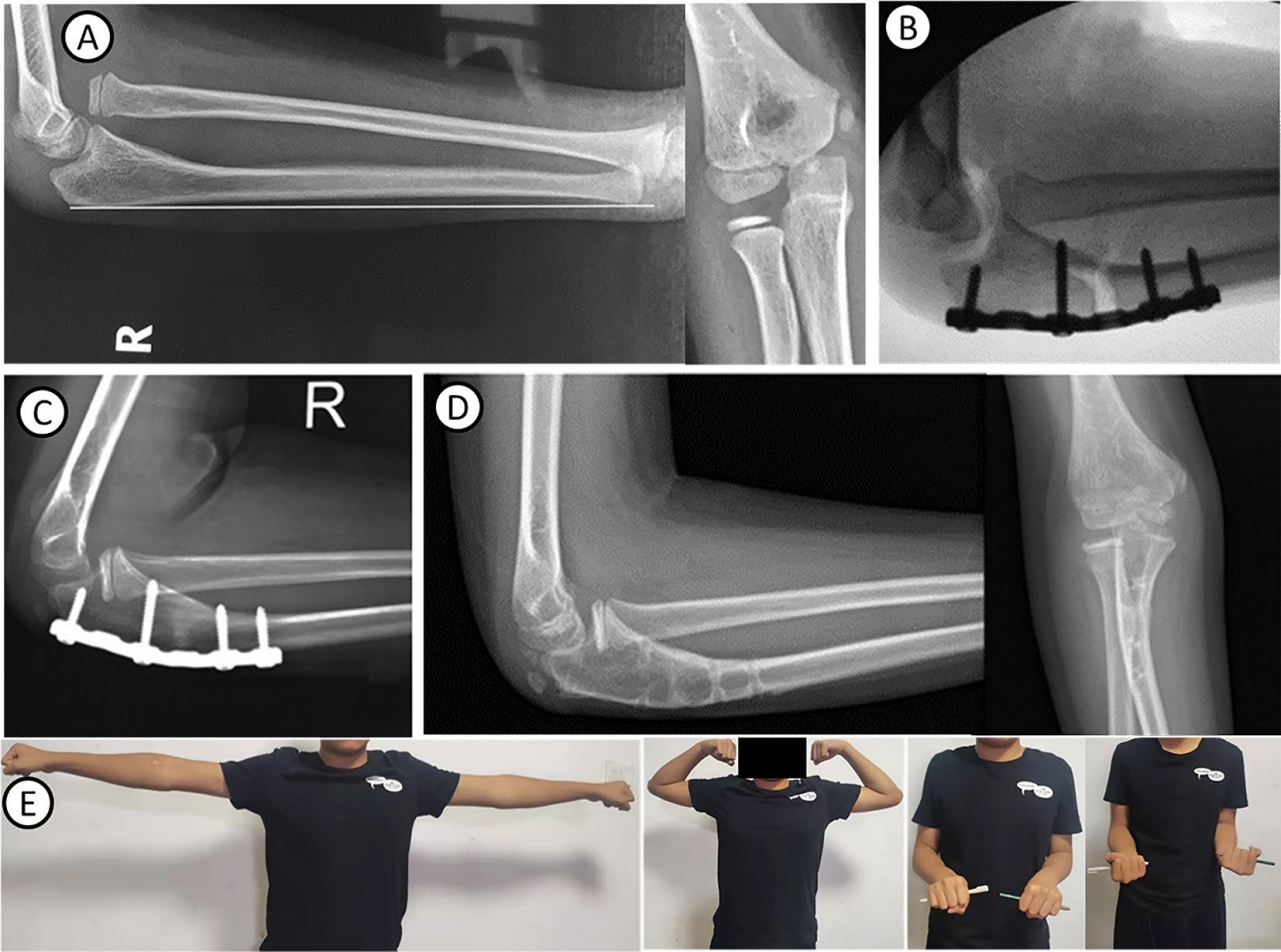
Typical case 2: An 8-year-old boy with right missed Monteggia fracture. (**A**) Bado type I, ulna bow sign is located at the distal end of ulna (group B). (**B**) Reverse angulation 12° during operation, radial head reduction. (**C**) At 5 months after operation, the radial head is in place, and the osteotomy end has completely healed. (**D**) At 1 year after operation, the radial head is in place. (**E**) The elbow joint function was good 4 years after operation.

The mean follow-up time was 33 months (range 6–102 months) in 28 children. The mean posterior angle of ulna during the operation was 21°. All postoperative incisions were healed in one stage. No delayed union or non-union was found at the osteotomy site. No loose or broken plate was found. Transcapitellar joint K-wire was used temporarily in 5 cases with unstable radial head reduction in surgical technique. In 2 of five cases, subluxation was observed within 2 weeks after the operation, and the patients were sent to the operating room for temporary fixation with K-wire of the radiocapitellar joint. Approximately 3–6 weeks after the operation, the anteroposterior and lateral X-ray results of the elbow joint were reviewed, the K-wire were removed at 3 weeks and the plaster cast were removed at 6 weeks. No K-wire breakage and pin tract infections was observed. The elbow joint functions of all children improved, especially the flexion and extension functions. The elbow flexion increased from 116° before the operation to 137° after the operation (P < 0.001). Forearm rotation function decreased. However, no statistical significance was noted (P > 0.05) (Table 2). Three children with radial nerve injury before operation were noted, and these patients underwent exploration and release during the operation. The finger extension function started to improve at 2–4 weeks and recovered completely at 3–4 months after the operation. X-ray results showed that the radial head was in place with no dislocation or subluxation and no delayed union or nonunion at the last follow-up. Typical cases are shown in Figs. 2, 3.

**Table 2:** Comparison of ROM (preoperative vs follow-up) in patients.

	Preoperative(°)	Follow-up(°)	P value
Flexion	116.07 ± 8.87	137.29 ± 5.48	0.000
Extension	0.82 ± 5.62	1.79 ± 3.92	0.471
Pronation	83.61 ± 2.56	80.29 ± 3.35	0.000
Supination	82.86 ± 2.92	82.46 ± 3.05	0.492

*ROM* range of motion.

### MUB and the osteotomy angle

**Figure 4:**
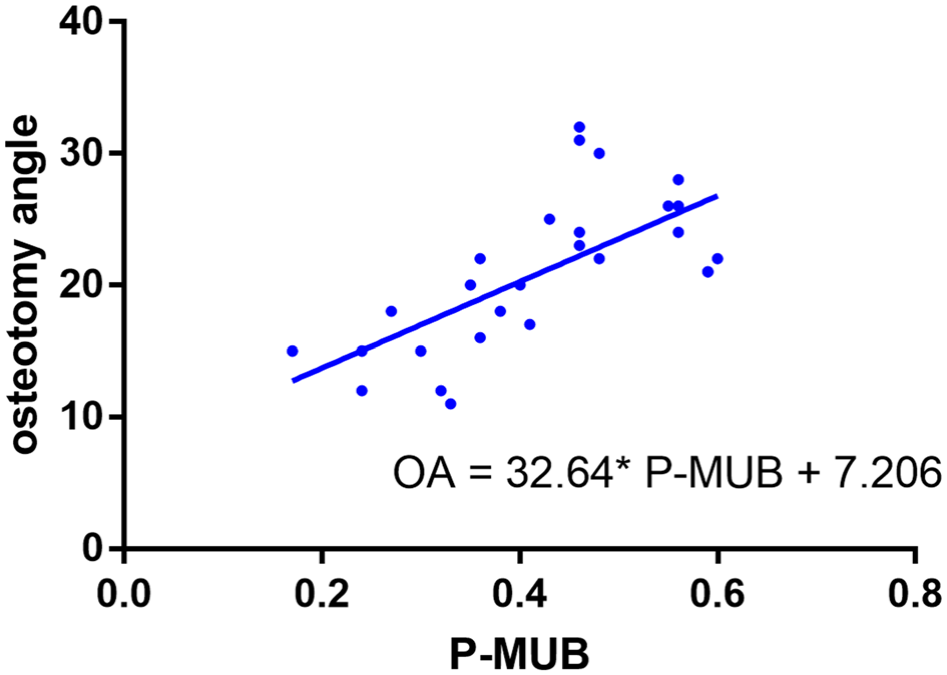
The linear regression equation. OA = 32.64* P-MUB + 7.206, R^2^ = 0.459, P < 0.0001.

The average R-MUB of 28 children was 0.036 (range 0.011–0.086), reaching 0.041 for group A (range 0.014–0.086) and 0.028 for group B (range 0.011–0.052). The P-MUB of group A was greater than that of group B. The average ulnar osteotomy angle of the 28 children was 20.93° (range 11°–32°); this was 24.64° (range 17.0°–32.0°) in group A and 15.60° (range 11°–22°) in group B (P < 0.001) (Table 3). A moderately correlation was found between the P-MUB and the osteotomy angle (OA) (r = 0.683, P = 0.000). A low correlation was found between the R-MUB and OA (r = 0.394, P = 0.038). Considering that the P-MUB had a good correlation with the OA, the P-MUB was used to calculate the linear regression equation of the OA; OA = 32.64* P-MUB + 7.206, R^2^ = 0.459, P < 0.0001 (Fig. 4).

**Table 3:** Comparison between two group in Intraoperative ulnar osteotomy angle and Post-operation ROM.

	Group A	Group B	P value
Intraoperative osteotomy angle	24.64 ± 4.47	15.60 ± 3.57	0.000
Post-operation flexion	136.59 ± 4.26	138.36 ± 6.87	0.412
Post-operation extension	1.88 ± 5.36	1.64 ± 3.53	0.875
Post-operation pronation	79.65 ± 3.67	81.27 ± 2.65	0.217
Post-operation supination	81.82 ± 3.03	83.45 ± 2.94	0.171

### Postoperative function

The flexion function of the elbow joint in each group improved significantly after the operation compared with that at pre-operation (P < 0.05) (Table 2). Group B exhibited a slightly better flexion, extension, and rotation functions than group A, but no significant difference was noted (Table 3). The flexion and extension functions of the two groups significantly improved. The rotation function slightly decreased, but no statistically significant difference was observed (Table 4).

**Table 4:** Comparison of two group in ROM (preoperative vs follow-up) in patients.

	Change in ROM (°)		P value
	A	B	
Flexion	+ 1.50 ± 7.29	− 0.80 ± 7.16	0.72
Extension	+ 21.29 ± 10.25	+ 21.09 ± 6.61	0.95
Pronation	− 3.59 ± 4.64	− 2.91 ± 4.99	0.72
Supination	− 0.76 ± 2.95	+ 0.18 ± 3.09	0.42

## Discussion

Type I Monteggia fractures are common and missed injuries in Chinese children^12–14^.

Numerous recent reports focused on the surgical treatment of Bado I type missed Monteggia fracture in children^4,7,8,15,16^. However, no study explored the influence of the relationship between ulnar bow sign and ulnar osteotomy angle on the surgical operation. Our research aimed to study the effect of the abovementioned relationship on the surgical treatment of Bado I type missed Monteggia fracture in children.

Bado type I Monteggia fractures caused by ulnar arch are most likely to be missed diagnosis and easily developed into chronic Monteggia fracture^10^. And the ulnar bow sign maybe a vital factor in measuring the injury^11^. Considering the rapid healing of children’s fracture, the fracture line disappeared after 3 weeks, and only the ulnar plastic deformation was left in the same direction as the radial head dislocation. The L-MUB and R-MUB need to be considered when designing the surgical plan of chronic Monteggia fractures. Hoon Park^11^ noted that when the largest ulnar bow sign is small and located at the distal end of the ulna, a stable reduction can be achieved without ulnar osteotomy. Satisfactory results were obtained by simple open reduction in seven cases of children whose ulnar bow was inconspicuous and located at the distal ulna. And Ulnar osteotomy is often needed when the MUB is greater than 3 mm and located at the proximal region. In the present study, the missed Bado type I Monteggia fractures in children were accompanied with an ulnar bow sign located in the area 20–60% from the distal ulna^11^. For areas within 40% of the distal ulna, the cases were grouped into two, namely, the middle and distal groups. In our study, we divided the group according to above research.

The location of the ulnar osteotomy had been explored in many researchers and most of them believed that it needed to be at the proximal end of the ulnar^17–19^. All patients in our study received ulnar osteotomy at the proximal end of the ulna. This setup is advantageous, because osteotomy at this location can induce sufficient tension in the interosseous membrane to align the radial head in the correct anatomical position. And interosseous membranes need to be preserved to avoid to limit forearm rotation^17,20–24^. In present studies, the rotation function of children after operation was slightly lost compared with that before the operation^8,12^. From our research, group B presented a better rotation function than group A, but no statistical difference was noted (Tables 2 and 3). It indicated a larger angle of ulnar osteotomy may tend to lost forearm rotation (Supplementary Information).

A prominent ulnar bow sign indicates the considerable severity of the interosseous membrane injury^25–27^. Our data indicated that the angle formation of ulna in proximal ulnar osteotomy was related to the P-MUB and R-MUB. When the position of the ulnar arch is closer to the middle of the ulna, the stable reduction of the radial head requires a larger angle ulnar osteotomy, as shown in Fig. 4. We consider that this is related to the degree of the interosseous membrane injury. When the position of the ulnar arch was in the middle of ulnar, the interosseous membrane was more serious injury. Therefore, it needs a greater degree of backward angulation after ulnar osteotomy to pull and reduce the radial head^5^.

Temporarily transcapitellar K-wire can be an efficient way in maintaining radiocapitellar joint^8,12^. However, most of surgeon do not use temporarily transcapitellar K-wire routinely. In our cases, only 5 patients were performed temporarily transcapitellar K-wire with larger P-MUB values, although we had increased osteotomy angle. In our opinion, reverse angulation of the ulnar osteotomy was the most important factor for radiocapitellar joint reduction. Temporarily transcapitellar K-wire was only a remedial measure when radiocapitellar joint was extremely instability. And two of the five cases showed subluxation within 2 weeks after operation, and were performed a second operation by temporarily transcapitellar K-wire. The reason for subluxation may be the following factors: Firstly, the longer time interval between injury and surgery or the older the child at the time of surgery. Nakamura et al.^5^ indicated that reduction over three years after injury and children older than 12 years has a poor prognosis. Secondly, due to the prolonged dislocation of the radial head, the annular ligament became thin and brittle after long-term compression by radial head^28^. Thirdly, because some children with missed Bado type I Monteggia fracture have a large value of P-MUB and R-MUB, the ulnar osteotomy angle required for good reduction of the radial head is increased, but it may not be enough in the operation.

## Conclusion

Our data showed that open reduction and ulnar osteotomy are reliable and effective methods for the treatment of missed Bado type I Monteggia fracture in children. The position of the maximum ulnar bow sign is an important parameter in surgical planning. When the ulnar bow is positioned at the middle ulna, a stable reduction of radial head needs to be achieved through a larger angle in the ulnar osteotomy.

## Supplementary Information

Supplementary Information.

## Data Availability

The datasets used and/or analysed during the current study available from the corresponding author on reasonable request.

## Abbreviations

MUB: Maximum ulnar bow
P-MUB: MUB position
R-MUB: The ratio of maximum ulnar bow
OA: Angle of ulnar osteotomy
